# Development of a Promising Bivalent Vaccine Against *Klebsiella pneumoniae* Based on Glycoengineered GMMA (geGMMA)

**DOI:** 10.1002/EXP.20240042

**Published:** 2025-08-03

**Authors:** Jingqin Ye, Wenhua Huang, Shujuan Yu, Yan Guo, Peng Sun, Ziyuan Chen, Linhui Hao, Yan Zhang, Caixia Li, Yongqiang Jiang, Jun Wu, Li Zhu, Hengliang Wang, Chao Pan

**Affiliations:** ^1^ State Key Laboratory of Pathogen and Biosecurity Beijing Institute of Biotechnology Beijing P. R. China; ^2^ State Key Laboratory of Pathogen and Biosecurity Institute of Microbiology and Epidemiology Academy of Military Medical Sciences Beijing P. R. China

**Keywords:** *Klebsiella pneumoniae*, geGMMA, vaccine, platform

## Abstract

Multidrug‐resistant *Klebsiella pneumoniae* constitutes a significant threat as a nosocomial pathogen, and no licensed vaccines are currently available. Generalized modules for membrane antigens (GMMA) have recently been recognized as a promising platform for developing outer membrane vesicle (OMV) vaccines against numerous infectious diseases. The study was carried out in use of the W3110 *ΔwbbH‐L ΔlpxM::lpxE* in which *E. coli* was treated in order to eliminate the endogenous polysaccharide and use two new ones (polysaccharides from *Klebsiella*). The exogenous polysaccharides were accurately displayed on the surface of spontaneously released OMVs. The immune responses evoked by subcutaneous administration of these vaccines were evaluated, and the protective effects were assessed using a mouse intraperitoneal challenge model. Interference in the biosynthesis of endogenous polysaccharides (such as deleting related gene clusters) is a viable approach to increasing the yield of glycoengineered GMMA vaccines (geGMMA). The geGMMA platform, which is conducive to safer large‐scale production, lays the foundations for the development of GMMA vaccines decorated with exogenous glycan antigens derived from pathogenic bacteria.

## Graphical abstract

Multidrug‐resistant *Klebsiella pneumoniae* poses a significant threat as a nosocomial pathogen, with no current licensed vaccines available. This study used a mutant *E. coli* strain expressing exogenous polysaccharides (from *K. pneumoniae*) to establish glycoengineered generalized modules for membrane antigens (geGMMA) vaccine platform, which demonstrated potent humoral immune responses and protection against *K. pneumoniae* infections. This approach could lead to safer large‐scale vaccine production and promote the development of GMMA vaccines with exogenous bacterial glycan antigens.

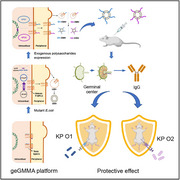

## 1 Introduction

*Klebsiella pneumoniae*, a well‐known human nosocomial pathogen, can lead to pneumonia, bacteraemia, sepsis, urinary tract infection, meningitis, and pyogenic liver abscess [1]. In recent years, the long‐term overuse of antibiotics has given rise to a surge in the incidence of multidrug‐resistant (MDR) *K. pneumoniae*, especially carbapenem‐resistant *K. pneumoniae* [2, 3]. Unfortunately, the slow pace of new antibiotic development lags behind the expanding population of drug‐resistant bacteria. Hence, vaccination has emerged as a robust defense against drug‐resistant bacterial infections. There are two categories of vaccines for *K. pneumoniae*: subunit vaccines (such as polysaccharides, conjugate vaccines, protein vaccines, and nanovaccines) and heat‐attenuated whole‐cell vaccines [4]. Considering the balance between safety and efficacy, nanovaccines have gradually become a preferred tool in vaccine design. Among the available options, the use of outer membrane vesicles (OMVs) is a promising strategy for vaccines.

OMVs are spontaneously released by many Gram‐negative bacteria during their growth and contain a wide range of pathogen‐associated molecular patterns [5], such as lipopolysaccharides (LPS), lipoproteins, and peptidoglycans, which confer self‐adjuvanticity [6]. In addition, the size of OMVs facilitates uptake by antigen‐presenting cells, aiding in presentation to T cells and follicular dendritic cells that activate high‐affinity antigen‐specific B cells, inducing an adaptive immune response. Given these advantages, OMVs have recently been acknowledged as a versatile platform for creating vaccines [7], and some OMV‐based vaccines have been licensed (such as 4CMenB for *Neisseria meningitidis* serogroup B and PRP‐OMPC for *Haemophilus influenza* type b). Moreover, OMV‐based vaccines for infections such as COVID‐19, invasive nontyphoidal *Salmonella*, *Neisseria gonorrhoeae*, and *Shigella*, are undergoing clinical development [7].

While successful examples of OMV‐based vaccines exist, some limitations must be addressed before their widespread application can be realized, especially improvements in safety and versatility. Generalized modules for membrane antigens (GMMA) have been reported to increase the OMV yield and reduce LPS endotoxicity [7]. Lipid A, an endotoxic component of LPS, can induce fever and potentially cause host death [8]. OMVs have been subjected to detergent extraction to reduce their endotoxic activity [9]. Genetic engineering is an alternative potent method for curtailing the reactogenicity of lipid A. Mutations were introduced to modify the lipid A structure, decreasing endotoxin levels, particularly by trimming the number of acyl chains and phosphate groups, which impacts the ability of lipid A to activate toll‐like receptor 4 (TLR‐4) and diminishes the inflammatory response evoked by lipid A [2, 6, 10, 11, 12]. Genes encoding acyltransferases from *Shigella, Salmonella*, and *N. meningitidis*, such as *htrB, msbB, pagP*, and *lpxL1*, have been mutated in the host to generate GMMAs with different penta‐acylated lipid A forms [13, 14, 15, 16]. GMMAs, as carriers of polysaccharides, such as those derived from *Serovars Typhimurium*, *Salmonella Enteritidis*, and *Shigella flexneri*, have been demonstrated superior to traditional glycoconjugate vaccines in animal models [17, 18, 19, 20]. Given these findings, we established a glycoengineered GMMA (geGMMA) platform by introducing an exogenous O‐polysaccharide (OPS) biosynthesis pathway.

In this study, we generated detoxified OMV‐based vaccines derived from an engineered *Escherichia coli* strain. A potential *E. coli* chassis cell was first obtained by reducing the reactogenicity of lipid A and eliminating the endogenous OPS biosynthesis gene cluster using CRISPR/Cas‐assisted multiplex automated genome engineering for rapid editing of the host genome [21]. Capsular polysaccharide and OPS are two prominent surface polysaccharides on *K. pneumoniae*. In contrast to the over 100 different structures of K‐antigens, O‐antigens can be divided into 11 unique serogroups [4]. Among them, the O1 and O2 serogroups account for over 50% of all antibiotic‐resistant *K. pneumoniae* strains [22]. Thus, the O1 and O2 antigens were selected as candidates. Then, the O1 or O2 polysaccharide biosynthesis pathway from *K. pneumoniae* was introduced into this engineered *E. coli* strain, enabling the display of the O‐antigen on the surface of spontaneously released OMVs. A series of animal experiments further demonstrated that these GMMA‐based vaccines could stimulate an excellent humoral immune response and offer robust prophylactic effects against *K. pneumoniae* infection. Notably, the bivalent vaccine also provided sufficient protection against challenges from *K. pneumoniae* serotypes O1 and O2 strains.

## 2 Results

### 2.1 Preparation and Characterization of Detoxified OMVs

**FIGURE 1:**
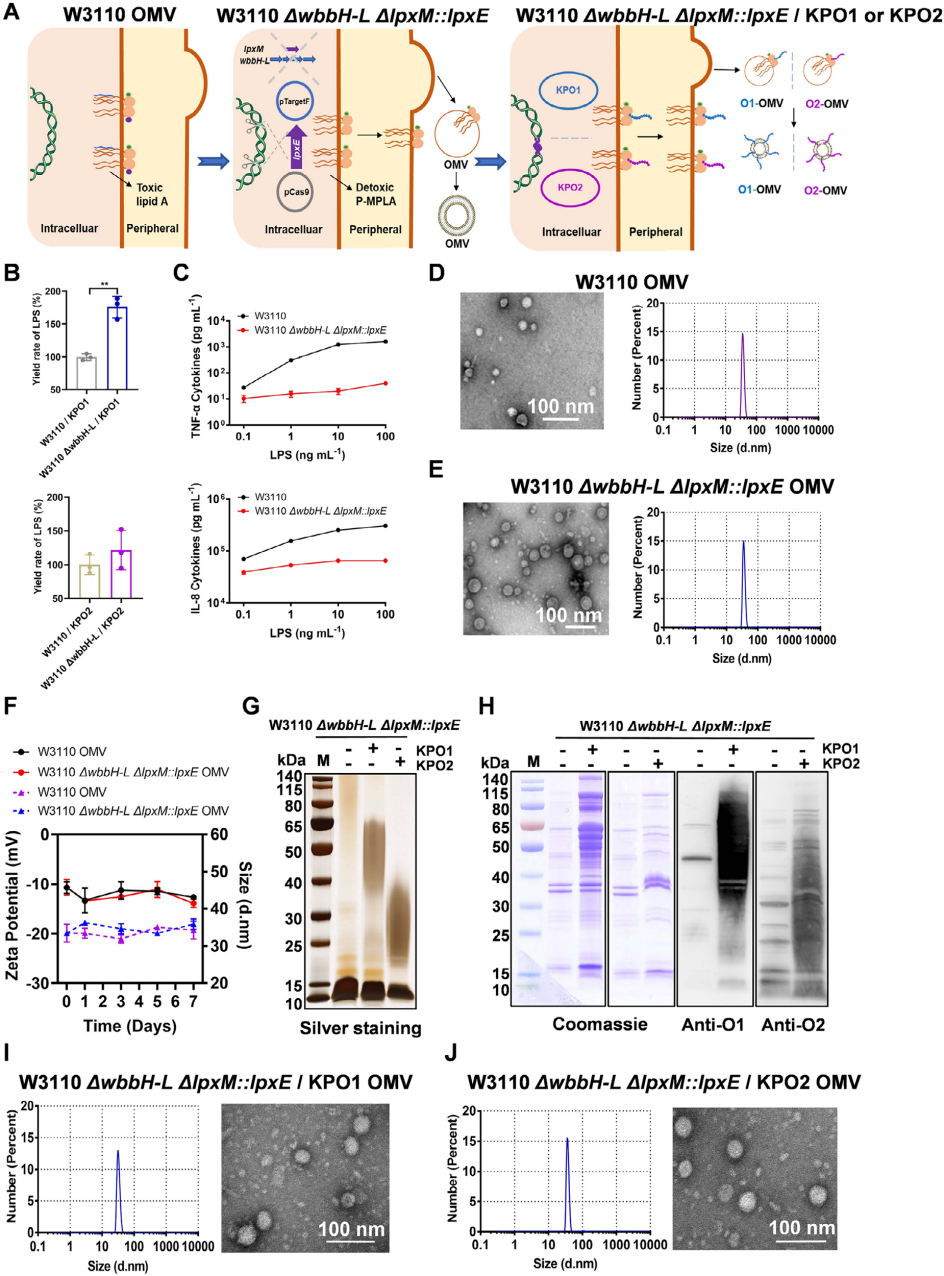
Construction and characterization of the OMV vaccine. (A) Schematic diagram of the geGMMA vaccine platform. (B) The yields of LPS from engineered *E. coli* (*n* = 3). (C) Cytokine production by THP‐1 cells after stimulation with LPS isolated from wild‐type or mutant *E. coli* strains (*n* = 4). (D) TEM images and DLS measurements of OMVs from wild‐type *E. coli* strains. Bar = 100 nm. (E) TEM images and DLS measurements of OMVs from mutant *E. coli* strains. Bar = 100 nm. (F) Stability analysis of OMVs from wild‐type and mutant *E. coli* strains by DLS (dashed line) and zeta potential (solid line) at different time points (*n* = 3). (G) Silver staining analysis of LPS from various strains. (H) SDS‒PAGE and western blotting analysis of detoxified OMVs, O1‐OMV, and O2‐OMV. (I) TEM images and DLS measurements of O1‐OMV. Bar = 100 nm. (J) TEM images and DLS measurements of O2‐OMV. Bar = 100 nm (** *p* < 0.01).

To maximize the synthesis of heterologous polysaccharide antigens, we first knocked out OPS synthesis‐related genes (from *wbbH* to *wbbL*) to suppress the expression of endogenous polysaccharides [23]. Then, we modified the structure of lipid A on the OMVs to reduce the inflammatory response. Following previous research [11], we constructed a detoxifying mutant of *E. coli* by deleting the acyltransferase gene *lpxM* and inducing the inner membrane phosphatase gene *lpxE* from *Francisella novicida* into *E. coli* to synthesize pentaacylated monophosphoryl lipid A (P‐MPLA). This mutated *E. coli* strain was named W3110 *ΔwbbH‐L ΔlpxM::lpxE* (Figure 1A; Figure S1). In our previous study, we successfully constructed *K. pneumoniae* O1 and O2 polysaccharide (KPO1 and KPO2) expression plasmids [23]. These expression vectors were introduced into W3110 and W3110 *ΔwbbH‐L*, and LPS was extracted from these *E. coli* strains. The increase in O‐antigen yield was assessed by comparing the yield (mg) ratio of LPS produced by 100 mL of whole‐cell bacteria before and after detoxification. The yield of O1 LPS increased by approximately 57% to 89%, and the yield of O2 LPS increased by 18% to 52% (Figure 1B) in the mutant strain, indicating an increase in yield after modification. After stimulating THP‐1 cells, which release a range of inflammation‐related cytokines [24], with LPS from either W3110 or W3110 *ΔwbbH‐L ΔlpxM::lpxE*, the levels of TNF‐α and IL‐8 were noticeably lower in the W3110 *ΔwbbH‐L ΔlpxM::lpxE* group than those in the control group, indicating decreased toxicity after modification (Figure 1C). OMVs were subsequently prepared by tangential flow filtration and ultracentrifugation methods. OAg expression levels of O1‐OMV and O2‐OMV were about 567.7 ± 77.4 and 665.9 ± 61.9 µg, respectively (indicating the mass of polysaccharide on the OMV obtained from 1 L bacterial supernatant). Transmission electron microscopy (TEM) revealed that the average diameter of the OMVs from strains W3110 and W3110 *ΔwbbH‐L ΔlpxM::lpxE* was 33 nm (Figure 1D,E). Dynamic light scattering (DLS) confirmed that the particles were consistent in size and had a uniform distribution (Figure 1D,E). For further stability analyses, the OMVs were exposed to a temperature of 37°C, and the size and zeta potential were monitored. DLS revealed that the OMVs maintained stability for up to 7 days after exposure to 37°C (Figure 1F).

The O1 and O2 expression vectors were subsequently transformed into W3110 *ΔwbbH‐L ΔlpxM::lpxE* to realize the expression of *K. pneumoniae* OPS on the OMVs (Figure 1A). After extracting LPS, silver staining revealed that the molecular weights of LPS with the O1 and O2 polysaccharides were approximately 50 and 30 kDa, respectively (Figure 1G). OMVs derived from W3110 *ΔwbbH‐L ΔlpxM::lpxE*, W3110 *ΔwbbH‐L ΔlpxM::lpxE /* KPO1, and W3110 *ΔwbbH‐L ΔlpxM::lpxE /* KPO2 were stained with Coomassie blue after SDS‐PAGE separation, and the presence of O1 and O2 polysaccharides was confirmed by western blotting (Figure 1H). TEM revealed that the average diameters of O1‐OMV and O2‐OMV were ≈35 nm (Figure 1I,J), which was slightly larger than that of OMV, likely due to the loading of polysaccharide antigens. The DLS results were consistent with the observed changes in size and showed a uniform distribution (Figure 1I,J), moreover, both O1‐OMV and O2‐OMV maintained stability for a week after exposure to 37°C (Figure S2).

### 2.2 Safety Evaluation of the Vaccines

**FIGURE 2:**
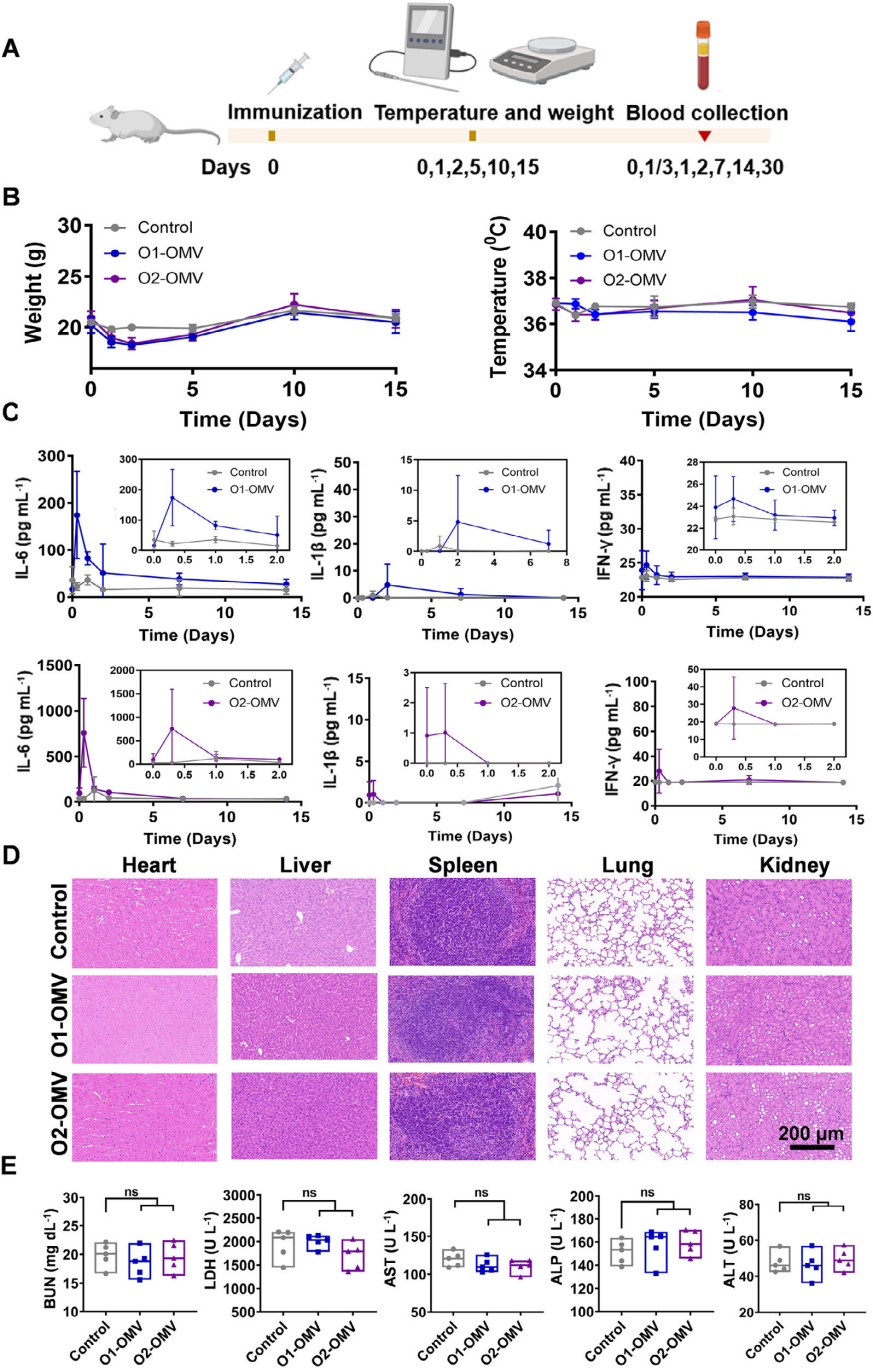
Safety estimation of OMV‐based vaccines. (A) Treatment schedule for the safety estimation experiment. (B) Temperature and weight changes in mice immunized with O1‐OMV or O2‐OMV (12.5 µg OPS per mouse) during the observation period (*n* = 5). (C) Cytokine levels (IL‐6, IL‐1β, and IFN‐γ) in the blood were measured at different time points post immunization (*n* = 5). (D) H&E staining analysis of the heart, liver, spleen, lung, and kidney on the 30th day post immunization. Bar = 200 µm. (E) Detection of serum biochemical indices, including BUN, LDH, AST, ALP, and ALT on the 30th day post immunization (*n* = 5).

After successfully constructing these two geGMMA vaccines, we performed a preliminary safety evaluation. BALB/c mice were immunized subcutaneously with O1‐OMV or O2‐OMV (containing 12.5 µg polysaccharide per mouse). A series of indicators were monitored continuously over the subsequent 30 days (Figure 2A). During the period of observation, the temperature of all the mice remained stable, and there was no difference in the change in body weight among the PBS, O1‐OMV, and O2‐OMV groups (Figure 2B). Cytokines (IL‐6, IL‐1β, and IFN‐γ) in the serum were detected at different time points, and all cytokine levels remained relatively low, particularly during the initial 2 days, indicating that the vaccines likely did not incite a systemic inflammatory response (Figure 2C). Additionally, haematoxylin and eosin (H&E) staining analysis conducted on the 30th day post‐injection revealed that the dissected heart, liver, spleen, lung, and kidney tissues of O1‐OMV‐treated or O2‐OMV‐treated mice were not obviously different from those of control mice (Figure 2D). Furthermore, the serum biochemical indices blood urea nitrogen (BUN), lactate dehydrogenase (LDH), aminotransferase (AST), alanine aminotransferase (ALP), and alanine aminotransferase (ALT) remained within the normal range on the 30th day following immunization (Figure 2E). These results demonstrate the remarkable safety and biocompatibility of these two geGMMA vaccines.

### 2.3 The geGMMA Vaccine Elicits an Effective Immune Activation

**FIGURE 3:**
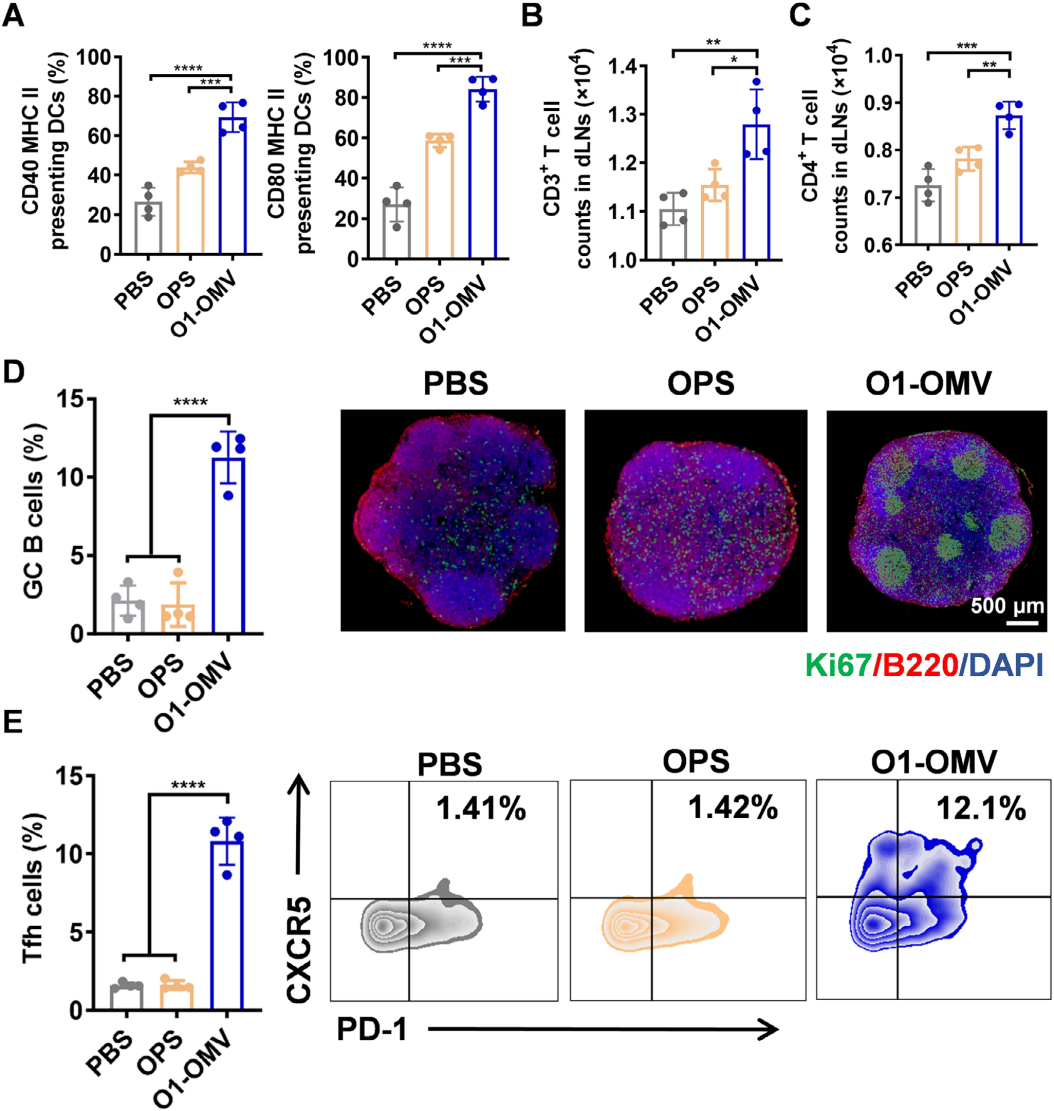
Evaluation of immune activation induced by O1‐OMV in dLNs. Evaluation of immune activation induced by O1‐OMV in dLNs. (A) Expression of the T‐cell recognition signal MHC‐II and costimulatory markers (CD40 and CD80) on DCs (MHC‐II^+^, CD40^+^, or CD80^+^ among the CD11c^+^ cell population) in dLNs 24 h after vaccination in mice (*n* = 4). (B,C) CD3^+^ T‐cell and CD4^+^ T‐cell counts in dLNs 3 days post vaccination (*n* = 4). (D) Proportion of GC B cells (GL7^+^ CD95^+^ cells among CD45^+^ cells) in the dLNs as determined by flow cytometry 7 days post vaccination (*n* = 4) and immunofluorescence image of dLNs stained with Ki67 antibody and B220 antibody 7 days post vaccination. Bar = 500 µm. (E) Proportion of Tfh cells (CXCR5^+^ PD‐1^+^ cells among CD4^+^ cells) in the dLNs as determined by flow cytometry 7 days post vaccination (*n* = 4). The data are presented as mean ± SD. Statistical analysis was performed using one‐way analysis of variance with Dunn's multiple‐comparison test (*p < 0.05, **p < 0.01, ***p < 0.001 and ****p < 0.0001).

The immune activation potential of the geGMMA vaccines O1‐OMV was evaluated by using PBS and OPS as controls. BALB/c mice were injected with these agents into the tail base subcutaneously. Some treated mice were euthanized 24 h post immunization, and their draining lymph nodes (dLNs) were extracted for flow cytometry analysis. The results indicated a significant increase in the number of DCs expressing the stimulatory markers CD40 and CD80, and the T‐cell recognition signal MHC‐II in the dLNs of mice immunized with O1‐OMV compared to those in all the other groups (Figure 3A). This distinction confirmed that the OMV‐based vaccine led to the most potent DC activation. The T‐cell distribution within the lymph nodes was further examined on the third day post immunization. Flow cytometry analysis of the dLNs revealed a notable increase in the total T‐cell (CD3^+^) and differentiated CD4^+^ T‐cell counts in mice immunized with O1‐OMV. Immunofluorescence analysis of the lymph nodes substantiated these observations (Figure 3B,C; Figure S3). In addition, we scrutinized the proportions of T follicular helper (Tfh) cells and germinal center (GC) B cells in dLNs after 7 days, considering that the affinity selection of GC B relies on CD4^+^ Tfh cells. Flow cytometry demonstrated a significantly higher proportion of Tfh and GC B cells in the O1‐OMV group than in the other groups (Figure 3D,E), suggesting a powerful immune response. Furthermore, GC formation in the dLNs was monitored through immunofluorescence. The results showed an increase in GC growth in mice injected with O1‐OMV, as determined by the detection of the proliferative markers Ki67 and B220 (Figure 3D). These findings illustrate that the geGMMA vaccine possesses a significant capacity to enhance the humoral immune response.

### 2.4 Protective Effect of the geGMMA Vaccine

**FIGURE 4:**
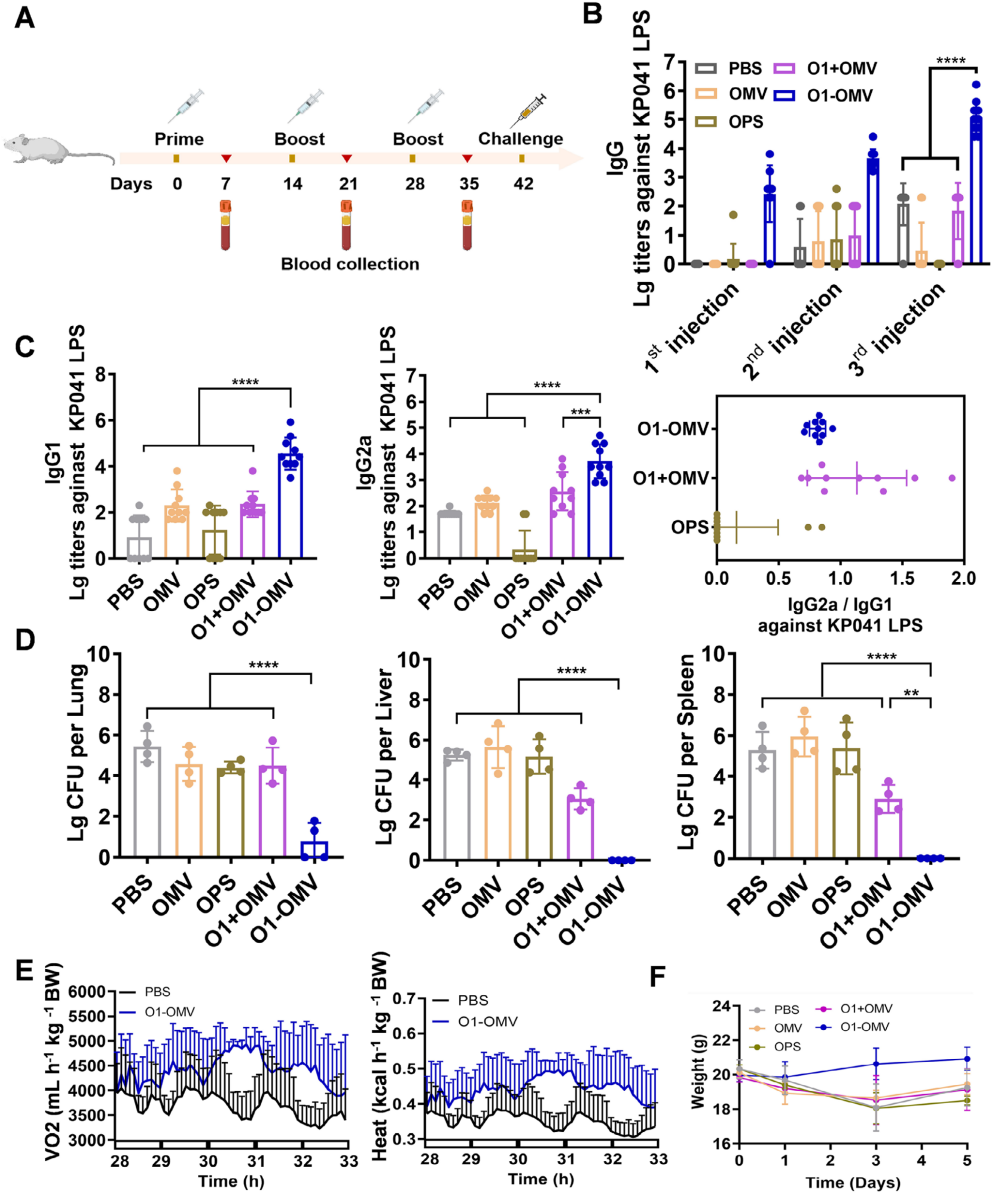
Evaluation of the strong antibody response and protective effect of the O1‐OMV vaccine. (A) Treatment schedule of the immunization experiment for titre evaluation. (B,C) IgG (B) and IgG subtype (IgG1, IgG2a) (C) titers against 041 LPS were measured in the serum of BALB/c mice immunized with OPS (indicated O1 polysaccharide), OMV, O1+OMV, or O1‐OMV after the third immunization. Analysis of IgG2a/IgG1 in the O1‐OMV group and other control groups (*n* = 10). (D) Bacterial loads in the lungs, livers, and spleens of mice after infection with the KP041 strain (*n* = 4). (E) Monitoring of energy metabolism 28–33 h post challenge in mice (*n* = 4). (F) Changes in the weights of the immunized mice after infection with KP041 (*n* = 4). The data are presented as mean ± SD. Statistical analysis was performed using one‐way analysis of variance with Dunn's multiple‐comparison test (*p < 0.05, **p < 0.01, ***p < 0.001, and ****p < 0.0001).

Encouraged by the remarkable immune‐activating ability of the O1‐OMV vaccine, we further evaluated the effect of the vaccine through a series of mouse experiments. BALB/c mice were injected subcutaneously with OMV, OPS, O1+OMV, O1‐OMV, or PBS on days 0, 14, or 28. Blood was collected for analysis from each mouse on days 7, 21, and 35 by tail snip (Figure 4A). ELISA‐based measurement of the IgG antibody titres against KP041 LPS (serotype O1) in serum samples on day 35 revealed an over 1000‐fold increase in O1‐OMV‐treated mice, which was significantly higher compared to all the other groups (Figure 4B). Further analysis of the IgG subtypes revealed significant increases in the IgG1 and IgG2a titres in mice immunized with O1‐OMV (Figure 4C), indicating that both humoral and cellular immune responses were enhanced when the polysaccharide antigen was expressed on the OMV carrier. Given that the IgG2a/IgG1 titre ratio typically serves as an indicator of potential Th1 or Th2 responses, we subsequently calculated the ratios and found that a mixed and balanced IgG2a/IgG1 systemic response was evoked by the OMV vaccine, indicating the activation of both Th1 and Th2 cells (Figure 4C).

The geGMMA vaccine candidate successfully elicited an OPS‐specific IgG antibody response, which prompted us to further evaluate its protective efficacy by intraperitoneal challenge with *K. pneumoniae* on day 42 (the 14th day after the third immunization, Figure 4A). Following immunization with one of five treatments (PBS, OMV, OPS, O1+OMV, or O1+OMV) as described above, each mouse was intraperitoneally injected with a dose of 1.5 × 10^4^ CFU of KP041 (GCA_902507275.1). Bacterial load analysis at 36 h after challenge in the lungs, livers, and spleens revealed a significant decrease in bacterial loads in mice immunized with O1‐OMV. The largest reduction was observed in the livers and spleens, in which almost no CFUs were detected, while at least 10^2^–10^3^ CFUs were counted in the other groups (Figure 4D). Moreover, histological analyses of the livers and lungs of KP041‐infected mice showed that, similar to the positive control group, KP041, immunization with O1‐OMV decreased inflammatory cell infiltration in the liver and sputum in the pulmonary bronchus, which also supported these results (Figure S4).

To observe physiological metabolic changes in the infected mice, oxygen and heat consumption were monitored between 28 and 33 h post challenge. The O1‐OMV‐immunized mice exhibited higher oxygen and heat consumption than did the PBS‐treated mice, indicating that the physiological metabolism of the immunized mice was maintained under more active physiological conditions (Figure 4E). Furthermore, the weight of each mouse after infection by *K. pneumoniae* was monitored for five subsequent days. The results indicated that the PBS‐, OMV‐, OPS‐, and O1+OMV‐treated mice exhibited continuous weight loss. Specifically, losses of approximately 10.9% (2.23 g), 7.2% (1.45 g), 11.2% (2.28 g), and 6.6% (1.3 g) were recorded by the third day. In contrast, the O1‐OMV‐treated mice demonstrated optimal results with minimal weight loss (Figure 4F). By detecting cytokines (IL‐6 and TNF‐α) at different time points, we found that the geGMMA vaccine could protect mice from cytokine storms (Figure S5). Taken together, these results demonstrate that O1‐OMV outperforms OPS, OMV, and O1+OMV in eliciting robust protection against *Klebsiella* infection.

### 2.5 Protective Effect of the Bivalent geGMMA Vaccine

**FIGURE 5:**
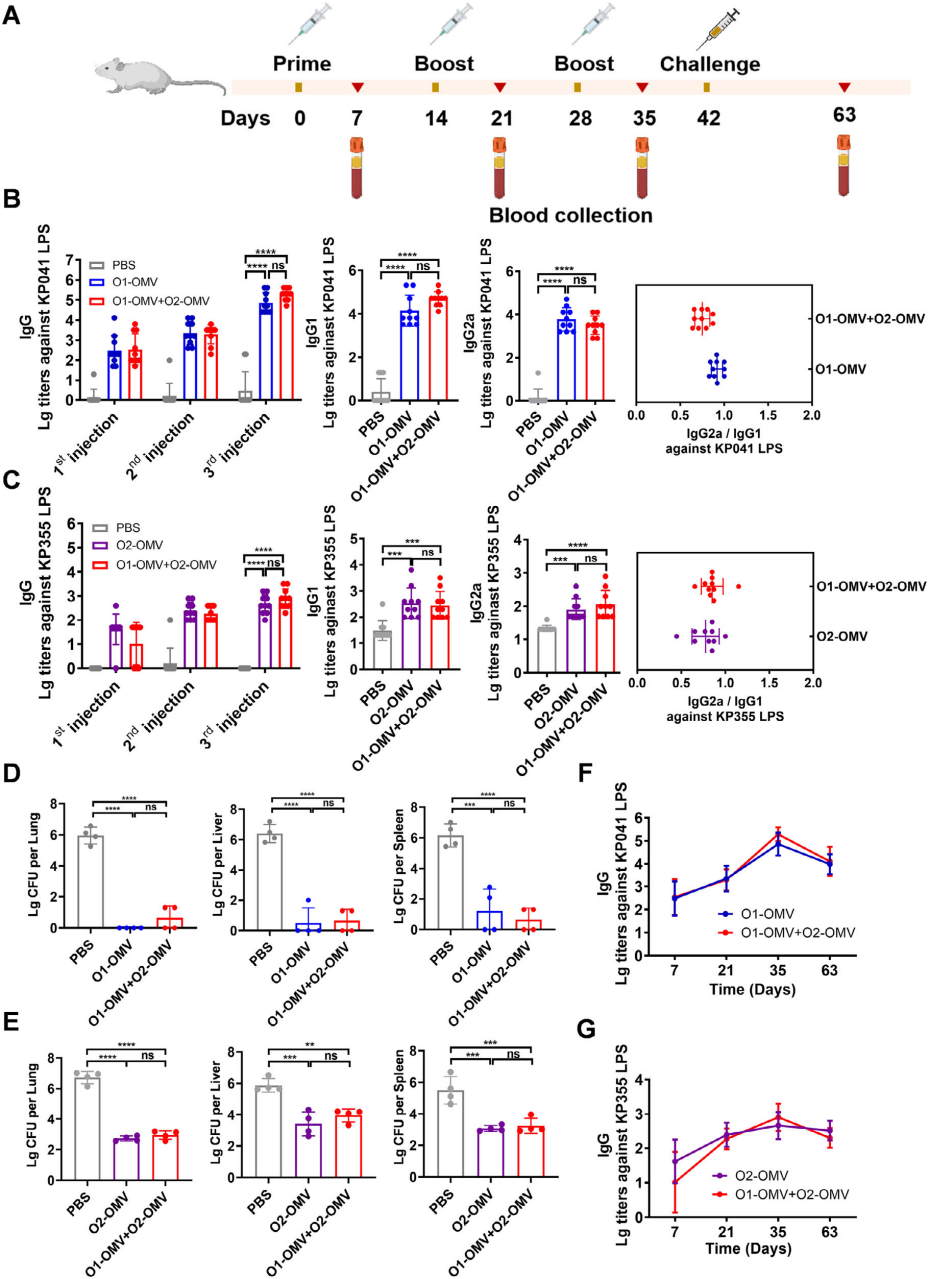
Evaluation of the strong antibody response and protective effect of the bivalent vaccine. (A) Treatment schedule of the immunization experiment for titre evaluation. (B,C) IgG and IgG subtype (IgG1, IgG2a) titres against 041 LPS (B) or 355 LPS (C) were measured in the serum of BALB/c mice immunized with O1‐OMV, O2‐OMV, or O1‐OMV+O2‐OMV after the third immunization. Analysis of IgG2a/IgG1 in O1‐OMV, O2‐OMV or O1‐OMV+O2‐OMV (*n* = 10). (D,E) Bacterial loads in the lungs, livers, and spleens of mice after infection with KP041 (D) or KP355 (E) (*n* = 4). (F,G) Long‐term evaluation of IgG titres in O1‐OMV‐, O2‐OMV‐ and O1‐OMV+O2‐OMV‐immunized serum (*n* = 10). The data are presented as mean ± SD. Statistical analysis was performed using one‐way analysis of variance with Dunn's multiple‐comparison test (*p < 0.05, **p < 0.01, ***p < 0.001, and ****p < 0.0001).

Encouraged by the obvious protective effects of the monovalent *K. pneumoniae* vaccine, we next constructed a bivalent vaccine by mixing O2‐OMV with O1‐OMV (O1‐OMV+O2‐OMV). BALB/c mice were subcutaneously immunized with PBS, O1‐OMV, O2‐OMV, or O1‐OMV+O2‐OMV on days 0, 14, and 28. Blood was collected from the tail vein on days 7, 21, 35, and 63 to quantify the antibody response to *K. pneumoniae* LPS. On day 42, the mice were challenged with *Klebsiella*, after which the bacterial load in various organs was monitored (Figure 5A). ELISA‐based measurements of the titres of the four antibody subtypes against KP041‐LPS in serum samples revealed increases in the O1‐OMV and O1‐OMV+O2‐OMV immunizations, but no differences were detected between these two groups (Figure 5B; Figure S6). Similarly, the evaluation of the four antibody subtypes against KP355 LPS (serotype O2) revealed an increase, but the difference was not significant between the O2‐OMV and O1‐OMV+O2‐OMV groups. (Figure 5C; Figure S7). The ratios of IgG2a/IgG1 indicated the activation of both Th1 and Th2 cells (Figure 5B,C).

The mice exposed to PBS, O2‐OMV, or O1‐OMV+O2‐OMV were treated with 4.0 × 10^7^ CFU of KP355 (GCF_903856825.1) via intraperitoneal injection on day 42 (14 days after the third immunization). The mice were euthanized 36 h post infection, and the bacterial loads in the lungs, livers, and spleens were quantified. Examination of these organs revealed that bacterial counts were lower in immunized mice than in their PBS‐treated counterparts when infected with either KP355 or KP041 (Figure 5D,E). These findings attest to the comprehensive protection afforded by the bivalent vaccine against both *K. pneumoniae* serotypes. Furthermore, the persistence of long‐term IgG titres induced by both bivalent and monovalent vaccines was noted. On day 63 (five weeks after the third administration), there was no significant decrease in the serum IgG titre in the mice treated with O1‐OMV, O2‐OMV, or O1‐OMV+O2‐OMV (Figure 5F,G), implying that these vaccines successfully stimulate enduring antibody responses for at least one month.

## 3 Discussion

Pneumonia is the most common cause of nosocomial respiratory tract infections and the second most frequent cause of Gram‐negative bacteremia [23, 25]. Many studies have shown increasing resistance of *K. pneumoniae* to antibiotics, resulting in an average rate of 1.63 outbreaks annually [26]. Here, we established a geGMMA vaccine platform against drug‐resistant *K. pneumoniae*. The exogenous polysaccharides of *K. pneumoniae* were expressed in the mutated *E. coli* by reducing the reactogenicity of lipid A and eliminating the endogenous OPS biosynthesis gene clusters to produce a GMMA vaccine. OMVs derived from detoxified *E. coli* are safer and easier to produce than OMVs from *K. pneumoniae* [27, 28, 29]. The spontaneous release of geGMMA simplifies subsequent purification and is conducive for large‐scale production. Moreover, the use of our system could be extended to other bacteria requiring strict and special culture conditions. In addition, OMV‐based vaccines against for other pathogenic bacteria could be prepared by simply replacing the target antigen biosynthesis pathways in this system.

Endogenous or exogenous antigens (derived from bacteria, viruses, parasites, or cancer antigens) have been displayed on the surface of OMVs [7]. Some local and systemic side effects of bacterial OMVs have been observed, such as disseminated intravascular coagulation and multiple organ dysfunctions, including in the heart. These safety issues limit the application of OMVs in the vaccine field [30]. Modification of the lipid A structure is an effective method for reducing unexpected reactogenicity. In this study, we inactivated the acyltransferase LpxM to remove the 3‐O‐acylation chain and introduced the inner membrane phosphatase LpxE to remove the 1‐phosphate group of lipid A in wild‐type *E. coli*, generating attenuated *E. coli* expressing low inflammation‐stimulated P‐MPLA (Figure 1A,C).

Increasing the yield of OMVs is another optimized direction through further deletion of *tolR*, *nlpI*, or other genes related to the stability of the linkage between the outer and inner membranes for overblebbing of the bacterial outer membrane. However, these vesicles did not exhibit uniform size distributions or morphologies. Therefore, we propose an alternative strategy, deleting gene clusters from *wbbH‐ to wbbL‐*encoded endogenous glycan biosynthesis to augment the expression of heterologous polysaccharide antigens in our geGMMAs. In addition, our OMV vaccine was approximately 35 nm in size, within the optimal range of 15–100 nm for direct delivery to dLNs [27]. Indeed, our vaccine showed good lymph node targeting ability and stimulated relative lymph node cell proliferation and differentiation. OMV‐based vaccines have a high ability to elicit polysaccharide antigen‐specific immune responses. Thus, low‐immunogenicity antigens such as OPS or peptides could be applied to the geGMMA platform.

Multiple antigen co‐expression has shown promise in the development of novel vaccines. OMVs produced by *E. coli* strains have been used as delivery systems for recombinant proteins. Proteinaceous antigens that fuse secretion signals or are expressed on a periplasmic surface have been used to express recombinant antigens in the lumen of vesicles [31, 32, 33, 34, 35, 36]. In addition, OMVs can serve as carriers of chemically or genetically linked polysaccharides, suggesting the possibility of exposing polysaccharides or proteins on vesicles directly [37], such as polysaccharide antigens from the *N. meningitidis serogroups* A and C, *H. influenza* type b, *Streptococcus* group A, and *Salmonella typhi Vi* [38, 39, 40]. The OMV secreted from Klebsiella contains various protein antigens (such as OmpA, OmpK35 and OmpK36) of itself [41, 42, 43, 44], resulting in stronger immune protection. However, considering the safety of vaccine preparation, detoxified manipulations should be needed in this strategy. That is, strain engineering needs to be done separately for each different serotype of bacteria. In our research, we have engineered an *E. coli* strain to mitigate biosafety risks during vaccine preparation processes. And the geGMMA platform established in *E. coli* has higher flexibility and could produce a series of pathogenic bacteria vaccines, targeting polysaccharide antigens of other pathogens, such as *Shigella spp*., *Salmonella spp*., *Yersinia spp*., et al.

With the development of ‘plug and play’ tools, many innovative tools have been applied to OMV vaccines. Catcher and Tag systems, such as SpyCatcher/SpyTag or SnoopCatcher/SnoopTag coupling, have been used in OMV‐based vaccines for tumor antigens [10]. Thus, other antigens with different properties could be added to our platform for designing multivalent vaccine platforms, such as those displaying HA from influenza viruses, the RBD from SARS‐CoV‐2, and other proteins or glycan antigens from pathogenic bacteria through coupling Catcher/Tag tools, modifying LPS, or fusing with the membrane proteins of OMVs [45].

In addition to the use of different antigen‐carrying methods, diverse administration methods are another advantage of OMV‐based vaccines. Traditional intramuscular administration does not provide a first line of protection against mucosal infection, especially in response to respiratory tract infections such as influenza or severe acute respiratory syndrome 2 (SARS‐CoV‐2), given the deficiencies in secretory IgA and IgG [46]. OMVs are good carriers that transport antigens to mucosal barriers. Hence, OMVs could be an ideal vaccine adjuvant with the capacity to elicit comprehensive immune responses and formulate various mucosal vaccines [47]. OMVs derived from *N. meningitidis* have been shown to carry the SARS‐CoV‐2 spike antigen, providing protection in mice and Syrian hamsters via intranasal and intramuscular delivery [45]. Similarly, intranasal immunization with OMVs has been found to be an effective route for protecting against *Acinetobacter baumannii* infection in mice [45]. Thus, our geGMMA platform is potentially suitable for mucosal delivery.

## 4 Conclusions

In summary, we constructed a detoxified geGMMA vaccine platform and prepared efficient bivalent OMV‐based vaccines against *K. pneumoniae*. Animal experiments illustrated that our OMV‐based vaccines could effectively elicit the humoral immune response and generate polysaccharide‐specific antibodies, thus providing outstanding protection against *K. pneumoniae* infection in sepsis models. Notably, the geGMMA platform also has promising potential for displaying multiple antigens.

## 5 Experimental Section

Experimental details are provided in the Supporting Information.

## Author Contributions

Jingqin Ye, Wenhua Huang, Li Zhu, Hengliang Wang, and Chao Pan designed the research. Jingqin Ye, Wenhua Huang, Ziyuan Chen, Linhui Hao, and Yan Zhang performed the experiments. Li Zhu, Hengliang Wang, Chao Pan, and Yan Guo supervised the study. Jingqin Ye, Shujuan Yu, Peng Sun, Caixia Li, Yongqiang Jiang, Jun Wu, Li Zhu, Hengliang Wang, and Chao Pan analyzed the data. Jingqin Ye, Li Zhu, Hengliang Wang, and Chao Pan drafted the paper. Jingqin Ye and Wenhua Huang contributed equally to this work.

## Conflicts of Interest

The authors declare no conflicts of interest.

## Supporting information




**Supplementary File 1**: exp270070‐sup‐0001‐SuppMat.pdf

## Data Availability

All data related to this work are present in the article and in the Supporting Information.
