# Supplementary material for: Development of a Promising Bivalent Vaccine Against Klebsiella pneumoniae Based on Glycoengineered GMMA (geGMMA)
Source: Exploration (Beijing). 2025 Aug 3;5(5):20240042. doi: 10.1002/EXP.20240042 (PMC12561431; doi:10.1002/EXP.20240042)
Supplement: Supplementary file 1 — exp270070‐sup‐0001‐SuppMat.pdf [file EXP2-5-20240042-s001.pdf]

Supporting Information

Development of a Promising Bivalent Vaccine Against *Klebsiella pneumoniae* Based on Glycoengineered GMMA (geGMMA)

Jingqin Ye[#a], Wenhua Huang[#b], Shujuan Yu[a], Yan Guo[a], Peng Sun[a], Ziyuan Chen[a], Linhui Hao[a], Yan Zhang[a], Caixia Li[a], Yongqiang Jiang[b], Jun Wu[a], Li Zhu*[a], Hengliang Wang*[a], Chao Pan*[a]

a. State Key Laboratory of Pathogen and Biosecurity, Beijing Institute of Biotechnology, Beijing, 100071, PR China
b. State Key Laboratory of Pathogen and Biosecurity, Institute of Microbiology and Epidemiology, Academy of Military Medical Sciences, Beijing, 100071, PR China

# Jingqin Ye and Wenhua Huang contributed equally to this work.

* Correspondence: Li Zhu, Hengliang Wang and Chao Pan, State Key Laboratory of Pathogen and Biosecurity, Beijing Institute of Biotechnology, Beijing, 100071, PR China.

E-mail: jewly54@bmi.ac.cn, wanghl@bmi.ac.cn, panchaosunny@163.com

EXPERIMENTAL SECTION

Ethics

All animal experiments were approved and conducted per the institutional guidelines of the Academy of Military Medical Sciences Institutional Animal Care and Use Committee (approval ID IACUC-DWZX-2021-P016).

Preparation of OMVs

E. coli was cultured at 37 °C in 1 L of Luria−Bertani (LB) medium (containing 1% peptone, 0.5% yeast extract, and 1% NaCl). When the culture's optical density at 600 nm (OD600) reached 1.0, the supernatant was separated by centrifugation (8000 rpm at 4 °C for 20 min). Then, the supernatant component was filtered through a 0.45-μm filter (Jingteng, China) and then concentrated to about 25 mL through a 100-K ultrafiltration tangential flow filtration cassette (Sartorius, UK). After that, OMVs were collected by further ultracentrifugation (110, 000 × *g* for 4 h at 4 °C). Then, the collected OMVs were filtered through a 0.45-μm filter (Millipore, USA), resuspended in 1 mL of PBS. The total protein concentration of the OMV was detected using the BCA protein quantification kit (Thermo Fisher, USA).

Strains and plasmids

The strains and plasmids used in this study are listed in Table S1. The strains mutations in this study were performed by using clustered, regularly interspaced, short palindromic repeat (CRISPR) method. All of the primers used are listed in Table S2. All of the bacterial strains were cultured in LB or on solid LB media supplemented with 1.5% agar. 50 μg mL^{-1} of antibiotics (such as spectinomycin, ampicillin, kanamycin, or chloramphenicol) were added as needed.

Supplementary Table S1. *E. coli* strains and plasmids used in this study.

Strains and Plasmids	Genotype Descriptions	Reference
Strains		
E. coli W3110	W3110 wild-type strain	[1]
W3110 *ΔwbbH-L ΔlpxM::lpxE*	The wbbH to wbbL gene clusters and the *lpxM* gene were knocked out in the W3110 strain	This work
W3110 *ΔwbbH-L*	The wbbH to wbbL gene clusters were knocked out in the W3110 strain	This work
Plasmids		
pCas9	Plasmid expressed tool enzyme in the CRISPR system	[2]
pTargetF	Plasmid with N20 sequence in the CRISPR system	[2]
pACYC184-KPO1	Carrying O1 serotype polysaccharide biosynthesis gene cluster in pACYC184	[3]
pACYC184-KPO2	Carrying O2 serotype polysaccharide biosynthesis gene cluster in pACYC184	[3]

CRISPR method

The gene mutations were generated through the CRISPR method described by Jiang *et al.*[2] Briefly, the N20 sequence of the single guide RNA was designed using the online N20 design tool (http://crispor.tefor.net/),[4] and the designed N20 sequence was subsequently inserted into the constitutively expressed plasmid pTargetF using the NEBridge® Golden Gate Assembly Kit (NEB #E1602). The sequence was verified by sequencing. The upstream and downstream sequences from the deletion cluster were connected by using Golden Gate method. pCas9 plasmid was transformed into the target strain by electroporation. Then, the recombinant strains are cultured and 2% arabinose was added. one hour later, the competent cells were prepared. Then, the strains were transformed with constructed pTargetF and the recombinant template DNA fragment. After overnight culturing on the LB plate, the correct mutation was obtained through polymerase chain reaction (PCR) and subsequent sequencing (Table S2). Then, the pTargetF and pCas9 plasmids were lost as described by Liu *et al.*[5]

Supplementary Table S2. Primers

Name	Primer
wbbH-in-F	5'-GGGCCTTAAGAAATTGAGAGAATACTATGAT-3'
wbbH-in-R	5'-TTCAGTTAAATGACGCTACGTTGATTTTTCTAC-3'
lpxE-in-F	5'-TTCCAGGGTTTCAAAGATATCTTTAAGAAAACCAAACTGCACAACCACAA-3'
lpxE-in-R	5'-TGGCTACCAACCATGATACGGCTACCCGCGAAAACAACGATCAGCAGGTA-3'
lpxM-in-F	5'-TATCCAGGTGTATTGTTCTGGTCGCGGACC-3'
lpxM-in-R	5'-GGGCCGGAGAAAATTCAGCCGCG-3'

Preparation of LPS and OPS

LPS extraction was performed as described previously.[3] Briefly, the cells, collected by centrifugation, were resuspended in ddH$_2$O. Then, 90% phenol was added with equal volume. After vigorously shaking at 68 °C for 30 min, the mixture was centrifuged (8000 rpm for 20 min at 4 °C) and the supernatant was collected. Phenol in the supernatant was removed through dialysis in ddH$_2$O. Then, DNase (1:1000, Solarbio, China) and RNase (1:1000, Solarbio, China) were added to the dialysed sample. After incubating at 37 °C for 3 h, proteinase K (1:1000, Solarbio, China) was further added to the sample for incubation again (60 °C for 1 h). Then, the mixture was boiled for 10 min, and LPS was obtained by centrifugation at 4 °C (8000 rpm for 20 min). To obtain OPS, the LPS solution was added with glacial acetic acid to a final concentration of 1%. Then, after boiling water bath for 90 min, the mixture was cool to room temperature and the pH was adjusted to 7.0. Finally, the solution was centrifuged (40, 000 × *g* for 5 h at 4 °C) to collected the supernatant.

The quantification of LPS and OPS

The content of LPS or OPS was subjected to an anthrone-sulfuric acid colourimetric carbohydrate quantification in the following manner: 100 μL of each sample and 1 mL of anthrone-sulfuric acid solution (2 mg mL^{-1}) were mixed together thoroughly in a glass test tube. The tubes were boiled for 10 min followed by cooling down with an ice-water bath. The color intensity was measured at 620 nm in a spectrophotometer (TECAN-Infinite F50, Switzerland), and compared with standards of known carbohydrate concentration. The sugar standards were diluted from a stock sucrose of known concentration.

Silver staining

LPS samples were mixed with an equal volume of 2 × SDS loading buffer and boiled for 10 min. The samples were added and separated via SDS–PAGE. Each gel was immersed in a fixing solution, containing 40% ethanol and 5% acetic acid, and slowly shaken twice for 15 min. Then, each gel was incubated with sensitising solution, containing 7% sodium acetate, 0.2% sodium thiosulfate, 30% ethanol, and 0.25% glutaraldehyde, for 30 min. After three times washing with ddH_2O, the gel was putted in silver nitrate solution, containing 2.5% silver nitrate and 40 μL of formaldehyde per 100 mL solution, for staining. Next, the gel was washed again with ddH_2O twice (1 min for each time) and incubated in developer solution, containing 0.75% sodium carbonate, 2.8 μL 5% sodium thiosulfate, and 40 μL formaldehyde per 100 mL solution. Based on the degree of color rendering, the reaction was terminated by adding a termination solution, containing 1.46% of $EDTA{\cdot}2H_2O$). Finally, the gel was washed with ddH_2O three times (1 min for each time).

TEM

The samples were diluted to approximately 50 μg mL^{-1}, added to 200 mesh copper grids, and incubated for 1 min. The excess sample was removed using filter paper, followed by the addition of 2% uranyl acetate for 45 s before air drying. Subsequently, the sample was visualized using a Hitachi HT7700 microscope operating at 80 kV.

DLS

The sample concentration was diluted to about 100 μg mL^{-1}, and 1 mL of the sample was added into the sample pool for detection by the Zetasizer Pro instrument. Each sample was tested three times in parallel.

Culture and stimulation of THP-1 cells

THP-1 cells were recovered and cultured in culture medium, containing 45% RPMI 1640, 45% DMEM, and 10% foetal bovine serum (Gibco, USA) at 37 °C with 5% CO_2. THP-1 cells (1 mL at 2×10^6 cells mL^{-1}) were plated in 24-well plates (Corning Costar, USA) and stimulated with PMA (100 ng mL^{-1}) for 24 h at 37 °C with 5% CO_2. After cell adhesion, the medium was replaced with fresh medium containing dispersed LPS at concentrations ranging from 0.1 to 100 ng mL^{-1} or

without added stimulus. After 24 h of incubation, the supernatants were harvested and stored at −80 °C until assayed. Cytokine levels in the supernatants were measured using commercial human IL-8 and TNF-α precoated ELISA kits (Dakewe Medical Equipment Co., Ltd., Shenzhen, China).

The preparation of O1 and O2 serum antibodies

Both O1 and O2 serum antibodies were prepared in our own lab. In detail, Japanese white rabbits were immunized twice on days 0 and 14 with inactivated bacteria mixed with Freund's complete adjuvant by subcutaneous injection. Then, each rabbit was immunized the third time on day 28 by subcutaneous injection of inactivated bacteria mixing with Freund's incomplete adjuvant. After that, each rabbit was immunized with inactivated bacteria (without adjuvant) on day 42 by intravenous injection into the ear margin vein. Three days post the last immunization, the heart blood of rabbits was taken, and the serum was isolated and absorbed by bacterial lysate before use. The inactivated bacteria were prepared as follows: The cultured KP041 (serotype O1, GCA_902507275.1) and KP355 (serotype O2, GCF_903856825.1) were respectively collected by centrifugation at 6, 000 rpm for 10 min at 4 °C, and then suspended and inactivated with formaldehyde in a final concentration of 0.5%. Japanese white rabbits (JW rabbit, female, approximately 2.5 kg) were purchased from Beijing Jinmuyang Experimental Animal Breeding Co., Ltd..

Coomassie blue staining and western blotting

OMVs were added with an equal volume of 2 × SDS loading buffer and boiled for 10 min. After being separated via SDS–PAGE, the protein in gels were stained with Coomassie blue, or transferred to PVDF membrane. Then, the membrane was incubated in blocking buffer, containing 5% skim milk in TBST (Tris-buffered saline with 0.05% Tween-20). After incubating at 37 °C for 1 h, the membrane was incubated with O1 or O2 serum antibody for 1 h. Then, the membrane was washed three times in TBST, and incubated with HRP-labelled goat anti-rabbit- antibody (TransGen Biotech, China, Cat# HS101-01) for 1 h, and subsequently washed three times in washing buffer. After adding a developing solution (Thermo Fisher Scientific, Waltham, USA), visualisation was performed on an Imaging System Tanon 5200 (Tanon Science & Technology, Shanghai, China).

Flow cytometry

dLNs were removed and the single-cell suspension was prepared for flow cytometry. The suspensions were incubated with APC-labelled anti-mouse CD3 (Invitrogen, USA, Cat# 17-0032-82,) and FITC-labelled anti-mouse CD4 (Invitrogen, USA, Cat# 11-0041-82) at 4 °C for 30 min in the dark. Likewise, for the analyses of Tfh cells and GC B cells, the single-cell suspension was prepared and stained with various flow cytometry antibodies, including FITC-labelled anti-mouse CD4 (Invitrogen, USA, Cat# 11-0041-82), APC-labelled anti-mouse CXCR5 (Biolegend, San Diego, CA, USA, Cat# 145511), PE-labelled anti-mouse PD-1 (Invitrogen, USA, Cat# 12-9985-81), APC-labelled anti-mouse CD45R (Invitrogen, USA, Cat# 17-0452-82), AF488-labelled anti-mouse GL-7 (Invitrogen, USA, Cat# 53-5902-82), and PE-conjugated anti-mouse CD95 (Biolegend, San Diego, CA, USA, Cat# 152607) at 4 °C for 30 min. Furthermore, to analyse the proportions of DC cells, the single-cell suspension prepared from dLNs was stained with various flow cytometry antibodies, including APC-labelled anti-mouse CD80 (Invitrogen, USA, Cat# 17-0801-82), FITC-labelled anti-mouse CD11c (Invitrogen, USA, Cat# 11-0114-82), PE-labelled anti-mouse CD40, and APC-A700-labelled anti-mouse MHC-II (Invitrogen, USA, Cat# 17-5321-82) at 4 °C for 30 min. After washing with staining buffer for three times, the cells were resuspended in 300 μL of staining buffer, filtered through a 200-mesh sieve, and analysed using CytoFLEX LX flow cytometer (Beckman Coulter Life Sciences, Brea, USA).

Safety evaluation

Before and after the subcutaneous injection of O1-OMV or O2-OMV, body temperature and weight of each mouse were detected on days 0, 1, 2, 5, 10, and 15. Blood was sampled at 0 h, 8 h, and 1, 2, 7, and 14 days from the tail vein, and the concentrations of cytokines (IL-6, IL-1β, and IFN-γ) in serum were detected by using ELISA kits (Dakewe Medical Equipment Co., Ltd., Shenzhen, China). 30 days post-injection, blood was taken, and the biochemical indicators (BUN, LDH, AST, ALP and ALT) in serum were determined by using a Chemray 240 automatic biochemical analyser (Rayto Life and Analytical Sciences, Shenzhen, China).

Mouse immunisation and challenge

Specific-pathogen-free six-week-old female BALB/c mice, purchased from Beijing Vital River

Laboratory Animal Technology Co., Ltd. (Beijing, China), were immunised three times on day 0, 14, and 28 by subcutaneous injection of 100 μL of PBS, OPS (from W3110 *ΔwbbH-L ΔlpxM::lpxE* / KPO1), OMV (from W3110 *ΔwbbH-L ΔlpxM::lpxE*), O1+OMV (mixing of OPS and OMV), O1-OMV (from W3110 *ΔwbbH-L ΔlpxM::lpxE* / KPO1), O2-OMV (from W3110 *ΔwbbH-L ΔlpxM::lpxE* / KPO2) or O1-OMV+O2-OMV (mixing of O1-OMV and O2-OMV) . The dose of OPS for each injection was 2.5 μg. Blood were sampled via a tail snip, and the serum were divided for further analyses. The mice were challenged intraperitoneally with encapsulated *Klebsiella pneumoniae* strains KP041 or KP355 (200 μL per mouse) two weeks after the third administration to evaluate the protective efficacy of the vaccines.

ELISA

Ninety-six-well plates were coated with KP041 LPS or KP355 LPS (10 μg well^{-1}). After incubating at 4 °C overnight, and washing three times with PBST, the plates were blocked with blocking buffer, containing 5% skim milk in PBST, at 37 °C for 2 h. Then, 100 μL of serum with serially dilution were added in each well and incubated at 37 °C for 1 h. Next, the plates were washed three times by washing buffer. 100 μL of HRP-labelled IgG (Abcam, Cambridge, UK, Abcam Cat# ab6820), IgG1 (Abcam, Cambridge, UK, Cat# ab97240), IgG2a (Abcam, Cambridge, UK, Cat# ab97245), IgG2b (Abcam, Cambridge, UK, Cat# ab97250), or IgG3 (Abcam, Cambridge, UK, Cat# ab97260), (1:15000) was added in each well, and the plates were incubated at 37 °C for 1 h. After washing three times with PBST again, color reaction was performed by using Soluble TMB kit (CWBio, Beijing, China). Finally, the absorbance at 450 nm of each well was tested using a microplate spectrophotometer.

Statistical analysis

The data were expressed as mean ± SD and statistical analyses were performed using GraphPad Prism version 8.0 (GraphPad, San Diego, CA, USA). The data are expressed as mean ± SD. The data were analysed by one-way ANOVA with Dunn's multiple-comparison test. Values of $p < 0.05$ were considered to indicate significance (**** $p < 0.0001$, *** $p < 0.001$, ** $p < 0.01$, and * $p < 0.05$).

Supplementary FIGURES

FIGURE S1

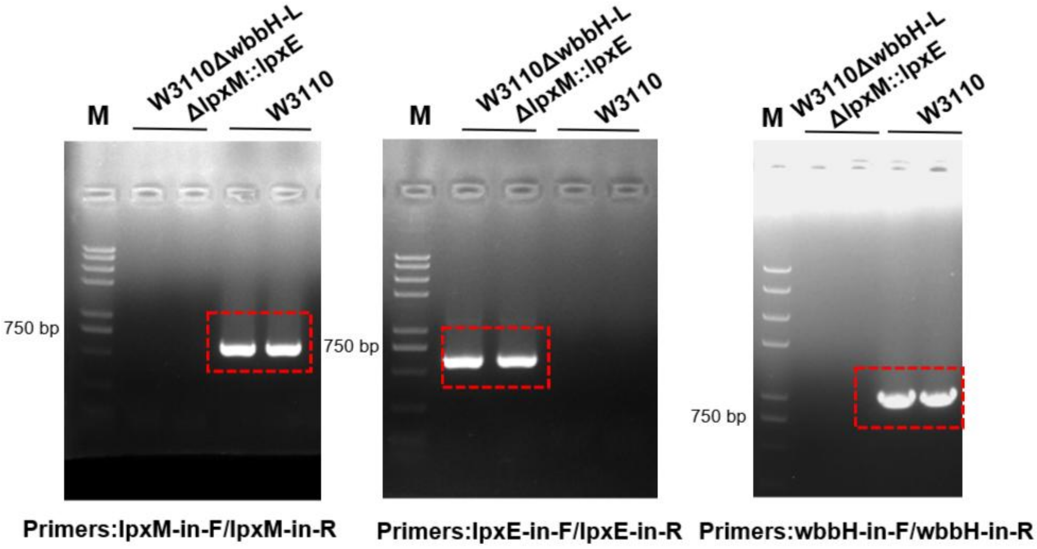

FIGURE S1 PCR validation of the constructed W3110 chassis strain (with deletion of the *lpxM* gene and the *wbbH* to *wbbL* gene cluster and introduction of the *lpxE* gene). WT3110 and W3110 *ΔwbbH-L ΔlpM::lpxE* strains were detected using lpxM-in-F/lpxM-in-R primers, lpxM-in-F/lpxM-in-R primers and wbbH-in-F/wbbH-in-R primers (nondeletion strains exhibited bands such as *lpxM* and *wbbH*, introduced strains exhibited bands such as *lpxE*).

FIGURE S2

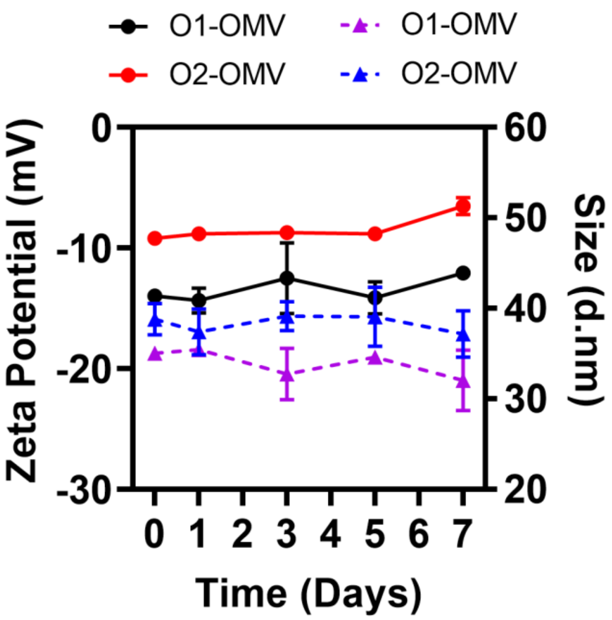

FIGURE S2 Stability analysis of O1-OMV and O2-OMV by DLS (dashed line) and zeta potential (solid line) at different time points.

FIGURE S3

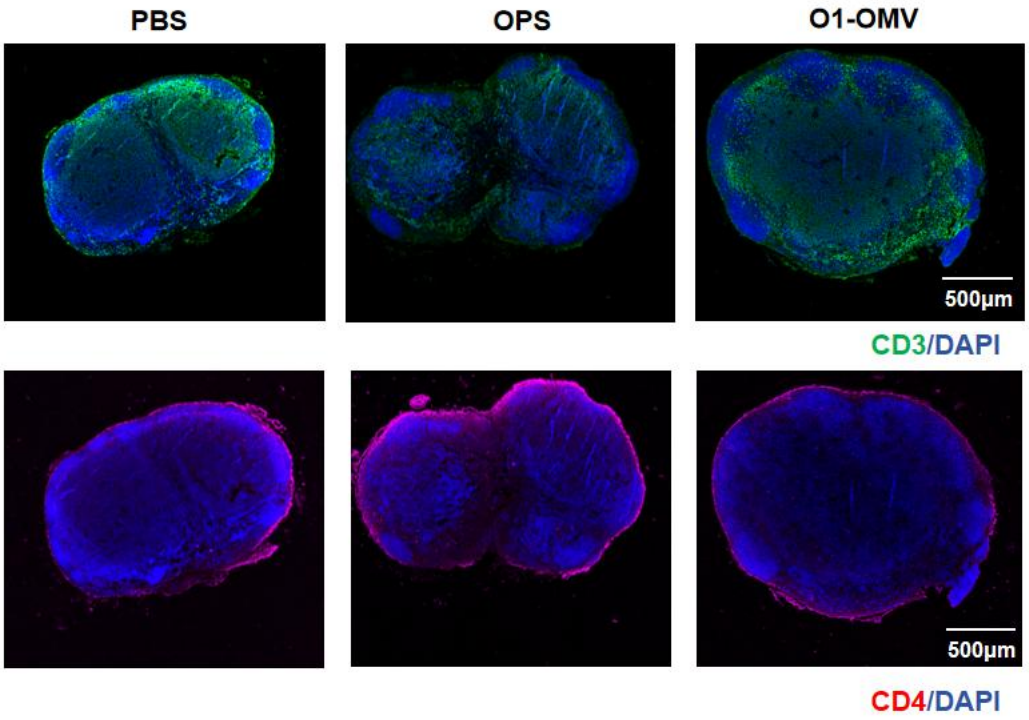

FIGURE S3 Immunofluorescence analysis of $CD4^{+}$ T cells in the dLNs at 3 days post immunisation. Bar = 500 µm.

FIGURE S4

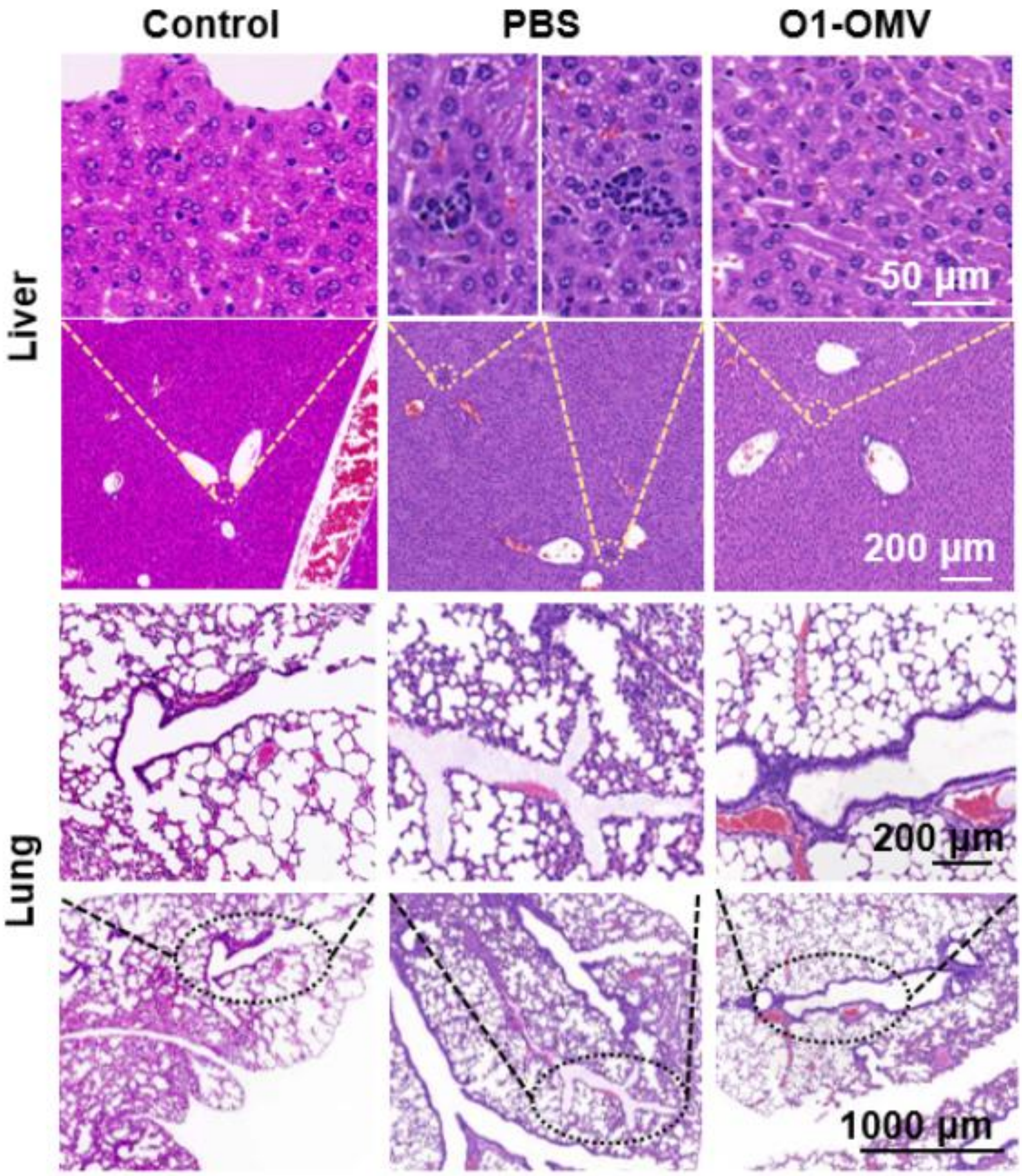

FIGURE S4 Histological analysis of the liver and lung tissues of infected mice and normal mice. At 36 h after infection with KP041, the mice were killed by cervical dislocation, and the liver and lung were subsequently dissected to observe pathological changes. The orange and black dotted boxes in the lower panels represent the fields of view in the corresponding figures above, and the scale bars are at the bottom of the figures.

FIGURE S5

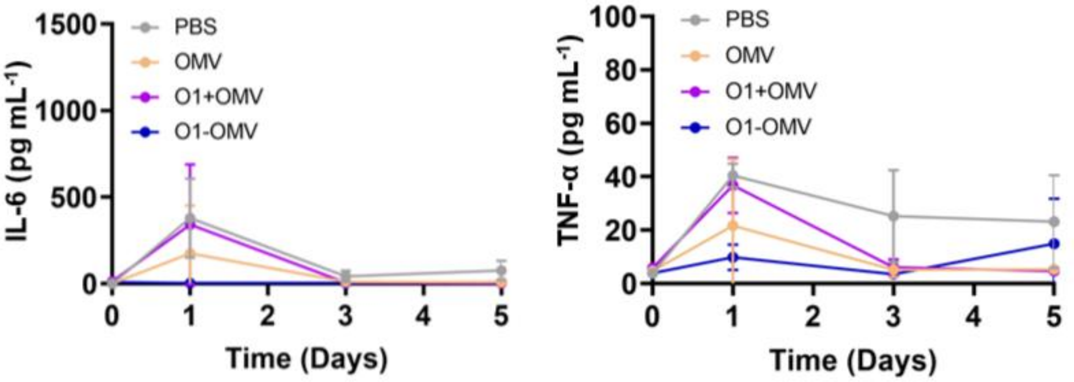

FIGURE S5 Cytokine levels of IL-6 and TNF-α in the blood at different time points post infection of KP041._(n = 3).

FIGURE S6

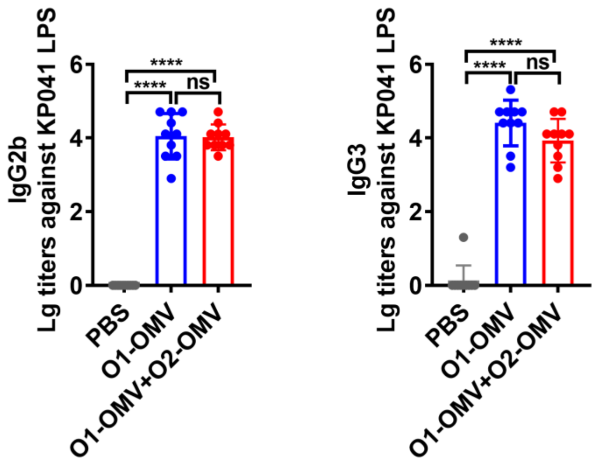

FIGURE S6 IgG2b and IgG3 subtype titres against KP041 LPS in the serum of BALB/c mice immunised with O1-OMV or O1-OMV+O2-OMV after the third immunisation (n = 10) (**** $p < 0.0001$, ns: no significance).

FIGURE S7

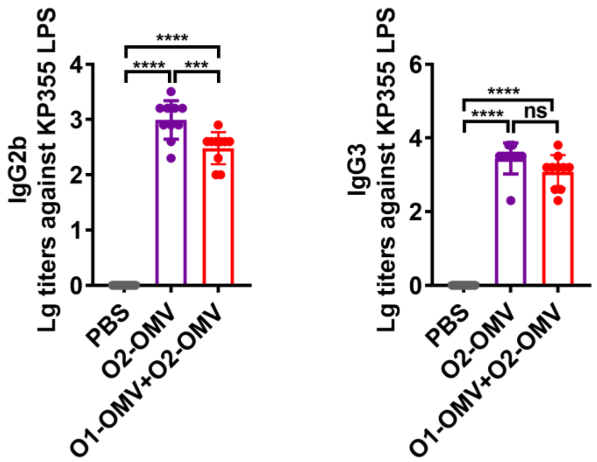

FIGURE S7 IgG2b and IgG3 subtype titres against KP355 LPS in the serum of BALB/c mice immunised with O1-OMV or O1-OMV+O2-OMV after the third immunisation (n = 10) (**** $p < 0.0001$, ns: no significance).

REFERENCES

[1] Y. Kohara, K. Akiyama, K. Isono, *Cell* **1987**, *50*, 495.

[2] Y. Jiang, B. Chen, C. Duan, B. Sun, J. Yang, S. Yang, *Appl Environ Microbiol* **2016**, *81*, 2506.

[3] P. Sun, C. Pan, M. Zeng, B. Liu, H. Liang, D. Wang, X. Liu, B. Wang, Y. Lyu, J. Wu, L. Zhu, H. Wang, *NPJ Vaccines* **2018**, *3*, 4.

[4] J. P. Concordet, M. Haeussler, *Nucleic Acids Res* **2018**, *46*, W242.

[5] Y. Liu, C. Pan, K. Wang, Y. Guo, Y. Sun, X. Li, P. Sun, J. Wu, H. Wang, L. Zhu, *Microb Cell Fact* **2023**, *22*, 95.
